# The relationship between presenteeism, quality of life and social support in higher education professionals: A cross-sectional path analysis

**DOI:** 10.1371/journal.pone.0267514

**Published:** 2022-04-21

**Authors:** Sónia Magalhães, Joselina Barbosa, Elisabete Borges

**Affiliations:** 1 Institute of Biomedical Sciences Abel Salazar, University of Porto, Porto, Portugal; 2 Faculty of Medicine of the University of Porto, Porto, Portugal; 3 Nursing School of Porto, ESEP, Porto, Portugal; 4 *Center* for Health Technology and Services Research–CINTESIS@RISE, Porto, Portugal; Universiti Pertahanan Nasional Malaysia, MALAYSIA

## Abstract

Presenteeism is the practice of being present at workplace, but not being able to carry out all the tasks due to health problems. Social support globally associated with health and wellbeing might positively influence presenteeism and consequently, the quality of life of these professionals. With this in mind, the aim of this study was to investigate the relationships between presenteeism, quality of life and social support in the work of non-teaching and non-research professionals within the context of higher education. A cross-sectional study was conducted, in which sociodemographic data were collected and the Portuguese versions of the Stanford Presenteeism Scale (SPS-6) (which includes the dimensions work-completed and distraction avoided) and Quality of Life Index (EUROSHIS-QOL-8) and the subscales of Supervisor’s Social Support and Peers’ Social Support of the Copenhagen Psychosocial Questionnaire (COPSOQ) were used. The questionnaire was applied online, and 322 professionals from a public university higher education institution in Northern Portugal participated in the study. Presenteeism was reported by 97 (30.1%) professionals. The peers’ social support was positively associated with quality of life. The supervisor’s social support was positively associated with distraction avoided and work completed and positively indirectly associated with quality of life, and the association was mediated by distraction avoided. We conclude that implementing strategies that can promote social support in the work context, namely strengthening networks between colleagues and competent and well-trained supervisors may prevent or reduce presenteeism in higher education professionals, as well as, provide a better quality of life.

## Introduction

Presenteeism, being the opposite of absenteeism, is generically defined as the practice of being present at the workplace but not being able to carry out all of the tasks due to health problems, with implications for the productivity of organisations and people’s health [1]. Presenteeism negatively affects work satisfaction and person’s wellbeing [2], in terms of health, performance and quality of life, as well as affecting the employer, in terms of productivity and consequent financial losses [3]. This situation worsens over the years of work [4].

The literature has shown that besides not being a recent phenomenon [1], it is clearly a globally observed phenomenon [3], which covers all sectors of activity, although it is more prevalent in the areas of care or welfare services, education and health [5]. While, for example, in the United States of America, research focuses more on the impact of the disease on productivity [6], in Europe it has focused more on the disease and the organisational conditions [1], with the latter being the basis for the development of the present study.

In Portugal, although there are few published studies on presenteeism, it can be observed in our institutions [7–9]. One of the most recent studies shows a 55% prevalence of presenteeism among Portuguese nurses [8]. This study also showed that the levels of presenteeism were less pronounced in young and less experienced nurses. In addition, female professionals were able to complete their work when they were ill than male professionals. Compared to other countries, Brazil (36%) and Spain (30%), Portugal had a higher presenteeism prevalence (55%) [8].

There are several health problems that may be at the root of presenteeism, from the least to the most severe, leading the latter to absenteeism and overall activity impairment [10]. These problems include chronic or episodic illnesses, such as seasonal allergies [11], headaches [11,12], musculoskeletal pains [12], gastro-intestinal problems [11], stress [10,11], depression [10] among others.

A review study points to a diversity of factors that may contribute to workplace presenteeism, ranging from personal (e.g., attitudes and orientations towards work, intrinsic motivation, feelings of satisfaction and accomplishment, involvement and commitment to work), to organizational (e.g., work overload, time pressure, lack of resources, job insecurity, availability of substitution, job demands, stress, strict absence management policies, competitive culture, limited promotion prospects), These factors appear to influence the decision to continue working even though ill [13], which results in a worse quality of life for the professional [14]. Regarding academic professionals, there has been evidence that the level of commitment to work is quite high [15]. This could mean that they would resist any interventions that would motivate them to withdraw from it, even when they suffer from serious illnesses. Hence, organisational factors outweigh personal ones [15].

With the emergence of the pandemic by COVID-19, presenteeism became an even bigger problem. Institutions had to adapt to remote working, teleworking, and embrace this new model as the norm. Virtual presenteeism, which means working virtually at home when you are ill, but not to the point of preventing you from working, has become a somewhat silent issue. At first, it is thought that autonomy and flexibility can help to reconcile work with personal and family life. However, it can have the opposite effect and lead to the compromise of the supposed balance. Continually working in the domestic space, with constant interruptions, makes it difficult to establish a boundary with work, which leads to excessive involvement in work activities [16,17]. Over-commitment to work can also lead to workaholism, which can hinder mental wellbeing and work-family balance [18].

In addition, after a period of confinement due to the COVID-19 pandemic, people resumed their work. Evidence suggests that in the post-pandemic phase (COVID-19), in the face of the precarious environment, workers chose to engage in more excessive work behaviours in order to protect their jobs and keep up with the demands of their jobs [16,17].

Although previous studies have significantly contributed to our understanding of presenteeism, it becomes essential to identify protective factors of this phenomenon that promote the positive functioning of professionals at the workplace and, consequently, improve their quality of life. Cumulatively, workers with the same health problems, despite presenting presenteeism, may reveal different levels of productivity loss. The characteristics of the professional and the organisational context interfere in presenteeism [19].

There is evidence that social support is positively associated with health and well-being at work [20]. In this context, social support can be an important factor in reducing presenteeism. Various sources of social support (e.g. informal support network), particularly the supervisor’s support, seem to be significant resources of health and well-being at work, and are considered key factors in promoting health at the workplace [20]. The supervisor’s social support and the establishment of a good relationship between leader and employee become relevant to decrease the levels of presenteeism and promote quality of life [21].

Working sick can also be considered an act of organisational citizenship, a sign of commitment and loyalty to employers and colleagues [19]. It is in this context that work can be beneficial, since it provides opportunities for involvement and social support, both of which are determining factors in coping with job stress and can replace or reinforce other absent resources, thus, work and personal resources can cushion the negative effects of work stressors, such as time pressure, work overload, and task uncertainty [20].

If supervisor support is lacking, colleagues can play a compensatory role in coping with difficulties (e.g., readiness to help and interpersonal relationships), as a protector of the negative impacts of presenteeism [17]. Social support from colleagues is termed as mutual help between co-workers in performing their duties, sharing both information and know-how, encouraging attitudes and camaraderie [22]. In this sense, social support refers to useful and available social interactions in the workplace from supervisors and colleagues [17,23].

Social support is the target of research in Portugal in organisational contexts due to its effects on quality of life. The same is not true for presenteeism.

Given the scarcity of studies on presenteeism, in Portugal, and the importance of this phenomenon in the organisational context, we believe that it is important to understand to what extent presenteeism and quality of life are related, as well as the influence that social support at work may have in these two domains. This will allow to equate adaptive strategies, not only of coping, but also of prevention.

Thus, the aim of this study is to investigate the relationships between presenteeism, quality of life and social support at work among non-teaching and non-researching professionals in university higher education.

## Materials and methods

### Study design and ethical standards

A cross-sectional study was designed and implemented in a Portuguese University School, in the North of Portugal. The study’s population consisted of all the University’s technical professionals. The data was collected between April and June of 2021 using a mailed self-administered questionnaire. The study was formally approved by the Ethics Committee of the Institute of Biomedical Sciences Abel Salazar, University of Porto, Porto, Portugal, and obtained a formally favourable opinion from the Data Protection Unit of the same University. All the participants were informed, before the survey, of the research’s purpose and the confidentiality principles. In addition, participants voluntarily signed informed consents online. They could withdraw from answering the questionnaire at any time.

### Measurements and data collection

A structured questionnaire was used in order to elicit information on participants characteristics, presenteeism, quality of life and social support. We collected data on demographic characteristics (age, sex, academic qualification, marital status, household) and labour characteristics (professional category, supervisor, labour contract, years of work at the institution, type of work and place of work (at home telecommuting or in the workplace)).

Presenteeism was evaluated using the Stanford Presenteeism Scale (SPS-6) which assesses the ability of workers to complete their work tasks despite health problems [24]. The literature shows that this is one of the most widely used instruments to measure presenteeism and confirmed good psychometric properties in the Portuguese population [7]. In this study the Portuguese version was adopted. The original scale [24] and version adapted to Portugal [7] discriminated two dimensions: completed work (CW) and avoided distraction (AD). The first factor focuses on the physical causes of presenteeism and corresponds to the amount of work done under the effect of the causes of presenteeism. The second factor is related to psychological aspects and corresponds to the amount of concentration mobilised to produce when there is a presenteeism effect. Each of these factors is assessed by three items, which totals six questions anchored on a scale with five response modalities (1- strongly disagree to 5- strongly agree) [7,24]. In the CW factor, the worst condition is to score "5—strongly disagree" on all three questions, indicating that the health condition interfered with work. In the AD factor, the worst condition consists of marking "1—I totally agree", where each numerical response value is converted into the opposite value. The total score of the SPS-6 corresponds to the sum of the values obtained in both dimensions. A higher value corresponds to a high level of presenteeism, i.e. higher performance at work. Health problems will be asked, in relation to the last month, based on the validation work of the scale for the Portuguese population [7].

Assessment of presenteeism was first calculated separately (each subscale) and then added together to obtain the global score. The resulting Cronbach’s α coefficient for this scale was 0.821 and for the subscales AD and CW it was 0.774 and 0.832, respectively, showing a good internal consistency.

Quality of life (Qol) was assessed using the Quality of life index (EUROSHIS- QOL-8) [25,26], an eight-item measure adapted from the World Health Organization Quality of Life–shortened version. The EUROHIS-QOL 8-index provides a generic measurement of quality of life covering four domains: physical, psychological, social, and environmental. This instrument showed good reliability and validity across a range of countries, and a universal one-factor structure with a good fit [27]. The psychometric properties of the EUROHIS-QOL 8-index validate its use in Portugal [26]. The internal consistency in the current sample was very good (Cronbach’s α = 0.829). Each item is answered through a Likert-type scale (five points), varying between "Very Bad" and "Very Good" or "Not at all" and "Completely" and also between "Very Dissatisfied" and "Very Satisfied". The sum of the eight items gives a total result, and a higher value corresponds to a better perception of the quality of life [25].

Social support at work was assessed using the Portuguese medium version of the Copenhagen Psychosocial Questionnaire (COPSOQ II) [28,29]. The COPSOQ is a validated and comprehensive questionnaire that gathers international consensus regarding its validity and comprehensibility in the evaluation of many of the most relevant psychosocial dimensions inherent to the work context [28]. The scales from the Interpersonal Relations and Leadership of the Portuguese medium version of COPSOQ II regarding Social Support from Supervisors (SSS) and Social Support from Colleagues (CSS) were used in this study. These scales are composed of three items each, assessed on a Likert-type scale (five points), ranging from "Never/almost never" to "Always". The score of the scales corresponds to the average of the respective items. A higher value corresponds to a high level of social support.

Assessment of social support was first calculated separately (each subscale) and then added together to obtain the global score (TSS). The resulting Cronbach’s α coefficient for this scale, TSS, was 0.857 and for the subscales CSS and SSS was 0.808 and 0.888 respectively showing a good internal consistency.

In this study, the total score of all scales was converted into a scale from 0 to 100.

### Statistical analysis

To summarize continuous variables, mean and standard deviation (SD) were used; for the categorical variables, absolute and relative frequency were calculated. T-test was used for comparison of study outcomes between groups.

The internal consistency of the scales was validated with Cronbach’s α coefficient.

The relationship between scores on the social support, presenteeism and quality of life were first examined using Pearson correlation analysis. To determine whether these significant correlations continued after controlling for confounding factors (age, sex, academic qualification, marital status, household, professional category, supervisor, labour contract, years of work at institution, type and place of work) backward stepwise multiple linear regression analyses were conducted. Finally, the relation between the study measures was examined through path analysis using the Maximum Likelihood method. Standardized regression weights were used to represent path coefficients between variables with p-values less than 0.05. Due to the exploratory nature of the model, we inspected the modification indices in order to see whether the addition of a new path would improve the overall fit of the model. To investigate the significance of indirect effects, this study used the bootstrapping method. A complete case population was used in this analysis. No variables showed values of skewness and kurtosis indicators of severe violations to the normal distribution. Model fit was determined by the following multiple indices: Chi-square statistic, comparative fit index (CFI values ≥ 0.9), Tucker Lewis Index (TLI values ≥ 0.9), root mean square error of approximation (RMSEA values ≤ 0.08) and PClose values ≥ 0.05 [30]. The significance level alpha was set to 5%. The software packages IBM SPSS Statistics 26.0 and AMOS 26.0 were used for the data analysis.

## Results

### Participants

There are 1658 employees of a public higher education institution in northern Portugal who were invited to participate in the study, of whom 325 (20%) fully responded to the sociodemographic questionnaire. Of these, three didn’t work last month and so they were excluded. According to the presenteeism scale criteria, from this group, only the ones who reported having gone to work ill in the last month were asked to respond to the second part of SPS-6. As a result, the final study population was built with a total of 97 (of 322) participants. This means that 30.1% professionals experienced presenteeism.

Table 1 shows the characteristics of the participants with presenteeism. The mean age was 46.1 (SD 8.4) and 71.1% were females. Most participants (60.8%) had higher school education and were married or are in an unmarried partnership (67.0%). Household consists on average of about 3 people (SD 1.1).

**Table 1:** Participants characteristics.

Participants Characteristics	N (%)
Sex	
Female	69 (71.1)
Male	28 (28.9)
Age, Mean (SD)	46.1 (8.4)
Academic Qualifications	
≤ 3^rd^ Cycle	12 (12.4)
Secondary	26 (26.8)
Higher Education	59 (60.8)
Marital Status	
Married or unmarried partnership	65 (67.0)
Divorced. Separated. Widow/er or Single	32 (33.0)
Household, Mean (SD)	2.8 (1.1)
Professional Category	
Operational Assistant	13 (13.4)
Technical Assistant	28 (28.9)
Senior Technician	53 (54.6)
Other	3 (3.1)
Supervisor	
No	82 (84.5)
Yes	15 (15.5)
Labour Contract	
Public	55 (56.7)
Private	42 (43.3)
Years of work at the institution, Mean (SD)	15.5 (8.7)
Type of Work	
Most physical	7 (7.2)
Most mental	53 (54.6)
Physical and mental	37 (38.1)
Last month worked	
Mostly or always at home, in telework	18 (18.6)
Same at home as at workplace	31 (32.0)
Mostly or always at workplace	48 (49.5)

Note: Data are presented as N *(%) unless otherwise indicated*.

Regarding participants’ labour characteristics, the group included 13 (13.4%) Operational Assistants, 28 (28.9%) Technical Assistants, 53 (54.6%) Senior Technicians and 3 (3.1%) had another category. Most of the participants were in a public contract (56.7%) and were not supervisors (84.5%). The average length of work at the institution was of 15.5 years (SD 8.7). The type of work was mostly mental work (54.6%) and in the last month 18.6% worked mostly at home, teleworking, while 32.0% worked half-half, at home and workplace, and 49.5% worked mostly at their workplace (Table 1).

#### Distribution and correlations of study variables

The presenteeism was self-declared by 97 (30.1%) workers, with a mean score of 50.0 (SD 21.3) on the global score of the SPS-6, ranging from 0 to 100. The CW dimension had a mean of 64.1 (SD 23.6). In AD the mean was 35.9 (SD 25.2). The mean score for TSS was 44.8 (SD 22.1), for CSS was 47.7 (21.7) and SSS was 41.8 (SD 26.0). The mean score for QoL was 49.2 (SD 15.4). Presenteeism, Social Support and Quality of Life are significantly and positively correlated (Table 2).

**Table 2:** Bivariate correlations and descriptive statistics of main study variables.

Outcomes	Mean (SD)*	1	2	3	4	5	6	7
1. AD	35.9 (25.2)	1						
2. CW	64.1 (23.6)	0.525*	1					
3. SPS-6	50.0 (21.3)	0.882*	0.864	1				
4. CSS	47.7 (21.7)	0.375*	0.299*	0.377*	1			
5. SSS	41.8 (26.0)	0.400*	0.325*	0.416*	0.710*	1		
6. TSS	44.8 (22.1)	0.413*	0.339*	0.432*	0.910*	0.938*	1	
7. QoL	49.2 (15.4)	0.537*	0.532	0.532*	0.478*	0.461*	0.509*	-

^a^ Score from 0 to 100.

* Significant at the p<0.05 level.

Among the participants who responded to the questionnaire but did not report presenteeism the mean score for CSS was 55.4 (SD 23.2), for SSS was 48.6 (24.4), for TSS was 52.0 (SD 20.0) and for QoL was 63.7 (SD 12.8) (S1 Table).

### Relations among presenteeism, quality of life and social support

To determine whether significant correlations continued after controlling for confounding factors stepwise multivariate linear regression analysis were conducted using social support and presenteeism domains to predict quality of life, adjusted to participants’ characteristics. Also, stepwise multivariate linear regression analysis was used to test the association between social support and presenteeism.

Quality of life remained significantly associated with AD (β = 0.267; p<0.001) and CSS (β = 0.217; p = 0.001). In turn, AD was associated with SSS (β = 0.331; p<0.001) and CW with SSS (β = 0.245; p = 0.006) (S2 Table). As a result, path analysis was used to evaluate the relationships between social support, presenteeism and quality of life at a multivariate level.

**Fig 1:**
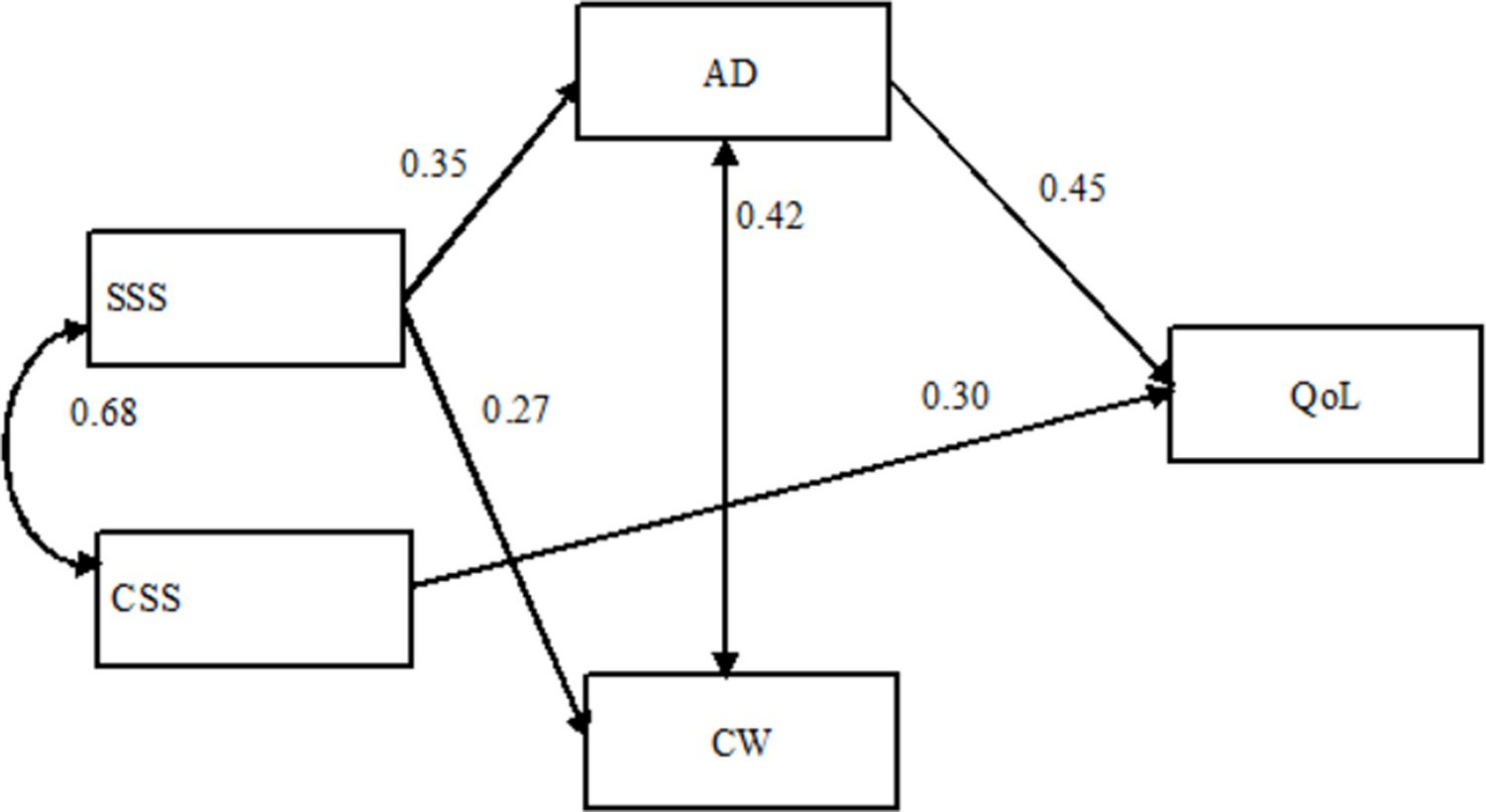
Path model depicting direct and indirect association between social support, presenteeism and quality of life. Values on single-headed arrows are standardized regression coefficients; values on the double-headed arrow are correlation coefficients. All paths are significant (p< 0.05).

Fig 1 shows the relationships proposed and the magnitude of effects among studied variables. The study model had good fit: X^2^(4) = 5.3; p = 0.262; CFI = 0.990; TLI = 0.976; RMSEA = 0.059, PClose = 0.371. Only significant trajectories were considered. CSS was not associated with AD or CW; in turn CW was not associated with QoL. SSS had significant positive association with AD (β = 0.35; p<0.001) and CW (β = 0.27; p = 0.009); CSS was positively associated with QoL (β = 0.30; p<0.001). Also, AD was positively associated with QoL (β = 0.45; p<0.001).

Table 3 shows the estimates of standardized direct, indirect and total effects of social support, presenteeism and quality of life. The indirect pathway from SSS to QoL (β = 0.15; p = 0.001) mediated by AD was significant.

**Table 3:** Summary of the direct, indirect and total effects of significant factors on social support, frailty and quality of life among haemodialysis patients.

Endogenous variables	Exogenous variables	Direct effect (p)	Indirect effect (p)	Total effect (p)
QoL	AD	0.45 (0.002)		0.45 (0.002)
	CSS	0.30 (0.003)		0.30 (0.003)
	SSS		0.15 (0.001)	0.15 (0.001)
AD	SSS	0.35 (0.002)		0.35 (0.002)
CW	SSS	0.27 (0.028)		0.27 (0.0028)

## Discussion

The aim of this study was to investigate the relationships between presenteeism, quality of life and social support at work in non-teaching and non-researching professionals in university higher education. The results showed that presenteeism was influenced by supervisor social support but not by peer social support. In turn, quality of life was directly influenced by peer social support and indirectly by supervisor social support through presenteeism.

Although literature shows that social support from colleagues and supervisors can minimise the influence of more demanding and competitive work situations [31,32], in our study only social support from supervisors was associated with presentism, not only in work completed, that is, the amount of work done under the influence of presentism that manifests itself from physical causes [7,24], but also in the avoided distraction, related to the professional’s capacity to complete the required tasks, and to remain focused despite the effects that presenteeism may have at a psychological level [7,24]. This result is corroborated by yet another study in health professionals showing that supervisor support had a positive effect on presenteeism, but that of co-workers did not [33]. There are authors who tell us that even if the social support of the colleague has a significant impact on distributive justice, the social support of the supervisor manages to have an even more relevant effectiveness in improving this justice because it is related to the allocation of rewards in an organization, considered very useful for the existence of balance effort-reward [33,34]. In this case, it can be said that high or low levels of perceived social support from colleagues do not promote presenteeism.

The literature refers us to the importance of the leader’s role, his/her ability to be attentive to the professionals’ needs, the respect for each worker’s limitations, adapting them to activities compatible with their work capacity [35], thus, with justification for adapting work to less serious health problems [36] and it is therefore important to support the redistribution of workloads and team planning [35,37]. Alertness is needed to prevent an insidious long-term deterioration in the health of the majority of the workforce, especially given the current COVID-19 pandemic context [36]. The associations between work characteristics and mental illness are well established [38]. Therefore, positive feedback from the supervisor is important as it leads to greater predictability, minimising role conflicts [37].

In addition, the results of the present study show that the relationship between quality of life and social support at work differ according to the type of social support. The relationship between supervisor social support and quality of life was fully mediated by distraction avoidance, i.e. workers with higher levels of supervisor social support may have higher levels of distraction avoidance, which may contribute to a better perception of quality of life. Within this framework, the evidence suggests that the supervisor’s social support functions as a moderator, being assumed as relevant for stimulating autonomy in work performance, and positive self-perception of well-being at work [32,39]. Despite the existence of presenteeism, which in this case leads us to the theory of a more positive side to it—can be synonymous with motivation at work—the latter can be conceptualised as beneficial for health, and convey a sense of significance [36,40]. Positively perceived organisational support leads to favourable psychological wellbeing, which has repercussions both on improving the quality of customer service and on job satisfaction [41].

A study with geriatric nurses reports that the fact that they do not consider their disease to be serious, or not aggravated because they choose to work, leads to an increase in presenteeism, which translates into a positive perspective. These professionals show satisfaction, pride and responsibility. Taking into account the organisational factors, they point out the incentive/remuneration policies, the connection between the work teams, the satisfactory interpersonal relationships; they also mention the personal factors related to a strong family support and the need to set an example by referring to the act of working [42].

In the case of social support from colleagues, this had a direct effect on quality of life, i.e. workers who have the support of colleagues perceive a better quality of life. This can be explained by the fact that social support from colleagues gives rise to social and confiding relationships, promoting personal well-being [43]. It is difficult to achieve thi s type of relationship with the supervisor, because he/she represents the organisation [44]. In this framework, the relationship between social support at work and quality of life is an important point arising from our study because it shows that the consequences of social support at work extend beyond the place where it is exercised. When social support at the workplace is high, quality of life outside the work environment also increases.

With regard to presenteeism, and based on the results obtained from the SPS-6 sub-scales, the score of 64.1 in the completed work dimension and 35.9 in the avoided distraction dimension, is synonymous with a higher level of presenteeism in one dimension compared to the other. Here, the psychological aspects are underlined [7,24]. There is a greater difficulty in concentrating and a higher psychological, rather than physical impairment [7,45,46]. This presupposes an intervention of the higher education organisations in the mental health of the professionals–otherwise, this might lead to an increase in the probability of occurrence of errors due to the performance of their functions with less ability to concentrate. It is necessary to consider that presenteeism, by being associated with the person who works, while feeling ill [14,47], worsens your quality of life [14,47,48].

Based on the sample of all professionals who participated in this study, we observed that participants without presenteeism obtained higher scores of social support and quality of life. Some literature defends that regardless of presenteeism being high or low, workers present worse health-related quality of life than those without presenteeism [14].

Implementing strategies that can promote social support in the work context, namely strengthening networks between colleagues, and having competent and well-trained supervisors, may prevent or reduce presenteeism among higher education professionals, as well as, provide a better quality of life. This context brings us back to policies aimed at increasing national well-being that should take into consideration the quality of working conditions and the factors that facilitate positive personal relationships [38]. In this sense, our target population could benefit from the implementation of a project based on the "3 in Line Programme" developed by the XXI Constitutional Government of the Portuguese Republic, which aims to promote a better balance between professional, personal and family life, and true equality between women and men to enjoy a citizenship that allows free choices in the various spheres of life. Otherwise, and as an example, there is evidence that poor health, the time spent at work, and the existence of presentism should be diagnosed and monitored by organisations in order to reduce the impact of work-family conflict. There are programmes that, for example, include flexible working hours, part-time work, and school holidays that make organisations more competitive in attracting and retaining professionals [49]. The importance of balance/conciliation is recognised as one of the fair working conditions and assumed in the “The European Pillar of Social Rights: An Assessment of its Meaning and Significance” [50] which, according to the literature, is a high-level political reaffirmation of social rights and principles. Its implementation is based on the entire governance acquis of the European Union established, for example, on regulations, funding actions and country-specific recommendations [51], that might be relevant contributions in the context presented in our study.

### Limitations

This study has some limitations. If on the one hand, the data collection may have been hampered by the pandemic period, this situation might be useful as a point of comparison for a post-pandemic period; on the other hand, the target population has been frequently asked to answer questionnaires and therefore there is some difficulty in having their support. This situation justifies the sample size and limits the generalisation and accuracy of our conclusions. Consequently, future studies should assess the relations of this study by professional class and focus on a longitudinal study. This would allow for a more specific intervention. In addition, and although this public university in the north of Portugal includes institutions in different areas of education, it would be useful in the future to expand the results to other organisations.

## Conclusions

The results obtained in this study provide valid information to university higher education institutions in terms of intervention to prevent or reduce presenteeism. We can conclude that social support at work is an important resource to deal with presenteeism and promote better quality of life. The supervisor’s social support can directly promote better levels of presenteeism and indirectly, quality of life, the latter being directly favoured by colleagues’ social support. Frontline supervisors should support professionals who attend work while ill, outlining clear goals and responsibilities so that professionals can focus on their priorities and remain effective despite health problems. On the other hand, taking into account the low value in the avoided distraction dimension, institutions should be more concerned with psychological aspects. This might increase awareness of the importance of implementing well-being measures. Specifically, we consider that the existence of counselling, social work and occupational medicine offices, working as a team, may plan a concerted intervention aimed not only at training supervisors, but also at preserving the health of the worker (with or without presenteeism).

## Supporting information

S1 Table Quality of life and social support among participants with and without presenteeism. (DOCX)

S2 Table Stepwise multivariate linear regression to predict associations between QoL, AD, CW, SSS and CSS. (DOCX)

S1 Dataset Study outcomes—Presenteeist participants. (PDF)
