# Supplementary material for: The relationship between presenteeism, quality of life and social support in higher education professionals: A cross-sectional path analysis
Source: PLoS One. 2022 Apr 21;17(4):e0267514. doi: 10.1371/journal.pone.0267514 (PMC9022867; doi:10.1371/journal.pone.0267514)
Supplement: S1 Table — (DOCX) [file pone.0267514.s001.docx]

**S1 Table.** Quality of Life and Social Support among participants with and without presenteeism

|  | Presenteeism | No-Presenteeism | p-value |
| --- | --- | --- | --- |
| CSS | 47.7 (21.7) | 55.4 (23.2) | 0.006 |
| SSS | 41.8 (26.0) | 48.6 (24.4 | 0.008 |
| TSS | 44.8 (22.1) | 52.0 (20.0) | 0.005 |
| QoL | 49.2 (15.4) | 63.7 (12.8) | <0.001 |
