# Supplementary material for: The relationship between presenteeism, quality of life and social support in higher education professionals: A cross-sectional path analysis
Source: PLoS One. 2022 Apr 21;17(4):e0267514. doi: 10.1371/journal.pone.0267514 (PMC9022867; doi:10.1371/journal.pone.0267514)
Supplement: S2 Table — (DOCX) [file pone.0267514.s002.docx]

**S2 Table.**  Stepwise multivariate linear regression to predict associations between QoL, AD, CW, SSS and CSS

|  | β (95% CI) | P value |
| --- | --- | --- |
| *Outcome:* QoL |  |  |
| CSS | 0.267 (0.095;0.339) | <0.001 |
| AD | 0.217 (0.157;0.376) | 0.001 |
| *Outcome:* AD |  |  |
| SSS | 0.331 (0.166;0.531) | <0.001 |
| *Outcome:* CW |  |  |
| SSS | 0.245 (0.068;0.418) | 0.006 |
