# Supplementary material for: The relationship between presenteeism, quality of life and social support in higher education professionals: A cross-sectional path analysis
Source: PLoS One. 2022 Apr 21;17(4):e0267514. doi: 10.1371/journal.pone.0267514 (PMC9022867; doi:10.1371/journal.pone.0267514)
Supplement: S1 Dataset — (PDF) [file pone.0267514.s003.pdf]

Presenteeist Participants	SPS-6			COPSOQ II			EUROSHIS- QOL-8
	AD	CW	Total	CSS	SSS	Total	
ID	Score	Score	Score	Score	Score	Score	Score
2	25,00	75,00	50,00	50,00	50,00	50,00	50,00
11	100,00	100,00	100,00	58,33	83,33	70,83	68,75
14	33,33	91,67	62,50	25,00	41,67	33,33	62,50
17	91,67	91,67	91,67	58,33	0,00	29,17	68,75
20	50,00	91,67	70,83	33,33	25,00	29,17	50,00
21	25,00	41,67	33,33	75,00	66,67	70,83	28,13
22	25,00	25,00	25,00	75,00	75,00	75,00	84,38
26	8,33	58,33	33,33	50,00	50,00	50,00	53,13
29	0,00	25,00	12,50	33,33	25,00	29,17	21,88
30	0,00	16,67	8,33	16,67	0,00	8,33	31,25
31	0,00	25,00	12,50	33,33	0,00	16,67	31,25
36	50,00	75,00	62,50	75,00	58,33	66,67	37,50
46	16,67	58,33	37,50	50,00	33,33	41,67	46,88
50	58,33	41,67	50,00	50,00	50,00	50,00	65,63
57	50,00	66,67	58,33	50,00	50,00	50,00	50,00
60	58,33	100,00	79,17	66,67	66,67	66,67	65,63
62	75,00	83,33	79,17	66,67	41,67	54,17	53,13
66	91,67	91,67	91,67	75,00	83,33	79,17	
73	50,00	75,00	62,50	33,33	25,00	29,17	59,38
77	100,00	100,00	100,00	50,00	50,00	50,00	
79	33,33	83,33	58,33	58,33	66,67	62,50	40,63
81	83,33	83,33	83,33	50,00	50,00	50,00	71,88
82	50,00	66,67	58,33	66,67	83,33	75,00	68,75
90	25,00	50,00	37,50	8,33	0,00	4,17	56,25
98	33,33	50,00	41,67	41,67	66,67	54,17	37,50
102	25,00	75,00	50,00	0,00	0,00	0,00	28,13
111	0,00	25,00	12,50	33,33	0,00	16,67	37,50
112	25,00	50,00	37,50	0,00	25,00	12,50	53,13
114				0,00	0,00	0,00	0,00
115	41,67	58,33	50,00	33,33	75,00	54,17	50,00
121	0,00	66,67	33,33	66,67	50,00	58,33	71,88
124	33,33	75,00	54,17	83,33	66,67	75,00	65,63
130	25,00	83,33	54,17	50,00	41,67	45,83	78,13
133	16,67	83,33	50,00	25,00	16,67	20,83	37,50
142	41,67	75,00	58,33	33,33	25,00	29,17	40,63
147	25,00	41,67	33,33	41,67	0,00	20,83	34,38
149	0,00	41,67	20,83	75,00	41,67	58,33	34,38
162	25,00	58,33	41,67	0,00	8,33	4,17	53,13
169	25,00	75,00	50,00	33,33	58,33	45,83	75,00
180	83,33	75,00	79,17	66,67	66,67	66,67	71,88
187	75,00	83,33	79,17	66,67	66,67	66,67	50,00
191	41,67	58,33	50,00	25,00	50,00	37,50	34,38
193	41,67	58,33	50,00	41,67	50,00	45,83	53,13
202	25,00	41,67	33,33	33,33	25,00	29,17	43,75
213	75,00	83,33	79,17	33,33	8,33	20,83	59,38
217	25,00	41,67	33,33	75,00	58,33	66,67	56,25
224	25,00	91,67	58,33	41,67	58,33	50,00	53,13

Presenteeist Participants	SPS-6			COPSOQ II			EUROSHIS- QOL-8
	AD	CW	Total	CSS	SSS	Total	
ID	Score	Score	Score	Score	Score	Score	Score
234	16,67	91,67	54,17	100,00	100,00	100,00	50,00
238	41,67	75,00	58,33	75,00	75,00	75,00	43,75
252	25,00	58,33	41,67	75,00	91,67	83,33	65,63
261	16,67	50,00	33,33	58,33	0,00	29,17	59,38
262	25,00	58,33	41,67	50,00	66,67	58,33	59,38
263	25,00	91,67	58,33	66,67	25,00	45,83	68,75
269	50,00	83,33	66,67	66,67	41,67	54,17	59,38
271	33,33	66,67	50,00	41,67	75,00	58,33	40,63
274	41,67	50,00	45,83	50,00	41,67	45,83	56,25
279	41,67	75,00	58,33	50,00	25,00	37,50	50,00
287	66,67	100,00	83,33	66,67	83,33	75,00	
294	58,33	58,33	58,33	83,33	66,67	75,00	62,50
301	66,67	75,00	70,83	66,67	50,00	58,33	40,63
302	0,00	16,67	8,33	50,00	25,00	37,50	31,25
305	41,67	33,33	37,50	16,67	16,67	16,67	46,88
306	25,00	50,00	37,50	25,00	50,00	37,50	43,75
307	8,33	41,67	25,00	25,00	25,00	25,00	31,25
312	66,67	75,00	70,83	41,67	25,00	33,33	43,75
313	25,00	75,00	50,00	33,33	25,00	29,17	50,00
315	33,33	66,67	50,00	33,33	33,33	33,33	62,50
317	0,00	33,33	16,67	25,00	25,00	25,00	28,13
318	16,67	41,67	29,17	33,33	16,67	25,00	37,50
319	25,00	75,00	50,00	50,00	50,00	50,00	50,00
320	8,33	25,00	16,67	25,00	16,67	20,83	31,25
323	25,00	83,33	54,17	91,67	83,33	87,50	43,75
324	50,00	75,00	62,50	50,00	33,33	41,67	40,63
325	0,00	100,00	50,00	33,33	25,00	29,17	25,00
326	33,33	75,00	54,17	58,33	33,33	45,83	62,50
329	0,00	8,33	4,17	16,67	0,00	8,33	25,00
330	25,00	58,33	41,67	41,67	41,67	41,67	53,13
331	75,00	100,00	87,50	75,00	91,67	83,33	
332	25,00	83,33	54,17	50,00	50,00	50,00	43,75
333	41,67	75,00	58,33	33,33	16,67	25,00	43,75
334	16,67	100,00	58,33	8,33	0,00	4,17	31,25
335	25,00	75,00	50,00	50,00	50,00	50,00	50,00
336	0,00	100,00	50,00	33,33	16,67	25,00	21,88
337	58,33	41,67	50,00	75,00	50,00	62,50	62,50
338	58,33	58,33	58,33	66,67	50,00	58,33	53,13
340	75,00	91,67	83,33	66,67	41,67	54,17	78,13
343	16,67	25,00	20,83	50,00	58,33	54,17	43,75
345	16,67	33,33	25,00	33,33	8,33	20,83	34,38
346	0,00	0,00	0,00	8,33	8,33	8,33	21,88
348	41,67	58,33	50,00	66,67	58,33	62,50	53,13
350	41,67	41,67	41,67	50,00	50,00	50,00	40,63
353	25,00	75,00	50,00	75,00	50,00	62,50	62,50
355	83,33	58,33	70,83	50,00	83,33	66,67	65,63
358	33,33	58,33	45,83	66,67	50,00	58,33	56,25

Presenteeist Participants	SPS-6			COPSOQ II			EUROSHIS- QOL-8
	AD	CW	Total	CSS	SSS	Total	
ID	Score	Score	Score	Score	Score	Score	Score
361	58,33	75,00	66,67	58,33	50,00	54,17	53,13
364	41,67	75,00	58,33	58,33	66,67	62,50	71,88
365	16,67	58,33	37,50	41,67	8,33	25,00	37,50
